# Elevation of iron storage in humans attenuates the pulmonary vascular response to hypoxia

**DOI:** 10.1152/japplphysiol.00032.2016

**Published:** 2016-07-14

**Authors:** Nicole K. Bart, M. Kate Curtis, Hung-Yuan Cheng, Sara L. Hungerford, Ross McLaren, Nayia Petousi, Keith L. Dorrington, Peter A. Robbins

**Affiliations:** ^1^Department of Physiology, Anatomy and Genetics, University of Oxford, Oxford, United Kingdom;; ^2^Department of Medicine, Royal Berkshire NHS Foundation Trust, Reading, United Kingdom; and; ^3^Nuffield Department of Medicine, University of Oxford, Oxford, United Kingdom

**Keywords:** hypoxia inducible factor, pulmonary hypertension, pulmonary circulation

## Abstract

Sustained hypoxia over several hours induces a progressive rise in pulmonary artery systolic pressure (PASP). Administration of intravenous iron immediately prior to the hypoxia exposure abrogates this effect, suggesting that manipulation of iron stores may modify hypoxia-induced pulmonary hypertension. Iron (ferric carboxymaltose) administered intravenously has a plasma half-life of 7-12 h. Thus any therapeutic use of intravenous iron would require its effect on PASP to persist long after the iron-sugar complex has been cleared from the blood. To examine this, we studied PASP during sustained (6 h) hypoxia on 4 separate days (*days 0*, *1*, *8*, and *43*) in 22 participants. On *day 0*, the rise in PASP with hypoxia was well matched between the iron and saline groups. On *day 1*, each participant received either 1 g of ferric carboxymaltose or saline in a double-blind manner. After administration of intravenous iron, the rise in PASP with hypoxia was attenuated by ∼50%, and this response remained suppressed on both *days 8* and *43* (*P* < 0.001). Following administration of intravenous iron, values for ferritin concentration, transferrin saturation, and hepcidin concentration rose significantly (*P* < 0.001, *P* < 0.005, and *P* < 0.001, respectively), and values for transferrin concentration fell significantly (*P* < 0.001). These changes remained significant at *day 43*. We conclude that the attenuation of the pulmonary vascular response to hypoxia by elevation of iron stores persists long after the artificial iron-sugar complex has been eliminated from the blood. The persistence of this effect suggests that intravenous iron may be of benefit in some forms of pulmonary hypertension.

## Abstract (precis)

*This study shows that a single dose of intravenous iron reduces the effects of hypoxia on the pulmonary circulation in a manner that persists for at least several weeks. This is long after the foreign iron-sugar complex has been cleared from the blood. It raises the possibility that manipulating iron stores, even in people who are not initially iron deficient, could be used for therapeutic gain in some forms of pulmonary hypertension*.

## NEW & NOTEWORTHY

*This study shows that a single dose of intravenous iron reduces the effects of hypoxia on the pulmonary circulation in a manner that persists for at least several weeks. This is long after the foreign iron-sugar complex has been cleared from the blood. It raises the possibility that manipulating iron stores, even in people who are not initially iron deficient, could be used for therapeutic gain in some forms of pulmonary hypertension*.

iron is a transition element essential to life. Healthy humans have a total iron content of ∼4 g, about half of which is incorporated into hemoglobin. As both Fe^2+^ and Fe^3+^ are stable in solution, iron is a highly reactive participant in oxidation-reduction reactions. This is exploited in many naturally occurring examples of catalysis, but also means that free iron is toxic, and consequently iron needs to be stored as a complex with macromolecules.

Oxygen acts as the terminal electron acceptor for aerobic metabolism. In large multicellular organisms, the flux of oxygen generates gradients in partial pressure within the organism, and these gradients are used to direct the development, maintenance, and physiological regulation of the systems for oxygen transport. Important within these regulatory mechanisms is the hypoxia-inducible factor (HIF) transcription system, which provides a coordinated cellular response to hypoxia.

At a molecular level, the regulation of iron and oxygen is tightly linked. Iron is an essential cofactor for the catalytic degradation of HIF by the prolyl-hydroxylases (PHDs), and furthermore HIF2α mRNA contains an iron-response element (IRE) within its 5′ end, so that the iron-regulatory proteins (IRPs) can regulate the expression of HIF2α (24, 39). In a complimentary manner, many of the proteins involved in the regulation of iron and iron storage are themselves HIF regulated (17). At the systems level, the importance of HIF to pulmonary vascular responses to hypoxia has been demonstrated both through experimental knockdown of the HIF pathway in mice (3, 38), which leads to abrogation of the rise in pulmonary arterial pressure in response to hypoxia, and through upregulation of the HIF pathway in rare human diseases and animal models of such diseases (4, 8, 27, 34), which leads to development of pulmonary arterial hypertension.

Taken together, the above findings suggest that iron availability itself may influence the pulmonary vasculature, and indeed this is the case. In humans, like hypoxia, iron chelation produces a progressive rise in pulmonary arterial pressure over a time period of 8 h (1), as indeed it does in circulating erythropoietin (EPO), which is a classical HIF-regulated protein (20). Importantly, administration of intravenous (IV) iron in humans abrogates the progressive rise in pulmonary arterial pressure that occurs in response to 8 h of sustained hypoxia (26, 33). In general, however, these experiments have focused on the response of the pulmonary vasculature to hypoxia on the same day as the iron administration. One exception to this is a study where IV iron was administered to participants who had already been at high altitude (4,340 m) for a period of 3 days and where the reduction in pulmonary artery systolic pressure (PASP) with iron was shown to last for a subsequent 4 days (28). However, these participants (both treated and control) will have had a cellular iron demand that was greatly increased through the enhanced rate of hematopoiesis associated with high-altitude exposure. Overall, it remains unclear whether the effects of iron on the pulmonary vasculature are arising as a relatively transient phenomenon, perhaps associated with the presence of an artificial iron-sugar complex in the blood, or whether they are effects that persist long after the elimination of the iron-sugar complex when the iron has been fully assimilated into the body's stores. Thus, while the close interaction between iron and the pulmonary vasculature has been well documented, the duration of effect of iron is unknown. Clinically, this is of importance as it will determine whether or not the effects of iron administration on the pulmonary vasculature will persist in time.

One area where iron administration may be beneficial is in some forms of pulmonary hypertension. In idiopathic pulmonary hypertension, iron deficiency is prevalent and carries a poorer prognosis (21, 23, 29). Supplementation of iron stores has been shown to be of benefit in patients with pulmonary arterial hypertension (22, 35), and further studies are underway to further evaluate this (10). In addition, recent studies have shown that iron deficiency is common in patients with chronic obstructive pulmonary disease, that the iron-deficient patients are more hypoxemic than their iron-replete counterparts, and that the prevalence of some degree of pulmonary hypertension is twice as great in the iron-deficient patients compared with those who are iron replete (16, 18).

The purpose of the present study was to determine whether, in healthy participants, the effects of iron loading on hypoxic pulmonary vasoconstriction persist long after the iron-sugar complex has been eliminated from the blood and when the iron has been incorporated into the body's stores.

## METHODS

**Fig. 1.**
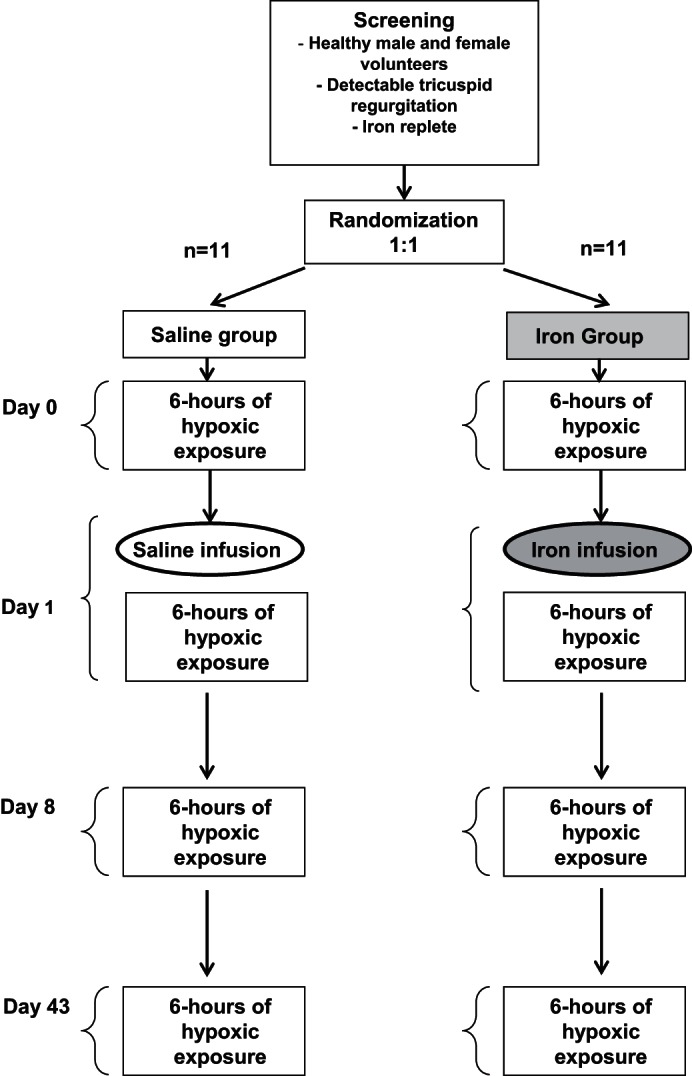
Protocol for the study. Twenty-two participants were recruited. Participants were block randomized 1:1 to iron or saline. Participants were exposed to sustained isocapnic hypoxia for 6 h on *days 0*, *1*, *8*, and *43*. *Day 0* was a baseline measurement day that was undertaken at least 3 days before *day 1*. An infusion of iron or saline was given on *day 1*.

The study protocol is illustrated in Fig. 1. Participants were block randomized (1:1) between two groups who were to receive either iron or saline infusions, respectively. There were 4 full study days (*days 0*, *1*, *8*, and *43*) in each limb of the study. *Day 0* was a baseline measurement day and occurred at least 3 days before *day 1*. On *day 0*, control measurements were made of the pulmonary vascular responses to a sustained (6 h) exposure to isocapnic hypoxia. On *day 1*, participants received either a saline infusion (NaCl 0.9%) or an infusion of 1 g of ferric carboxymaltose. Following this infusion, participants underwent a second 6-h exposure to sustained hypoxia to assess their pulmonary vascular response. These measurements were repeated again at 1 wk (*day 8*) and 6 wk (*day 43*) after the saline or iron infusion.

The study was conducted in a double-blind manner. The investigator who randomized the participants, administered the infusions, and collated the blood results was not involved in any other part of the study. The participants were blind to the nature of the infusion as they wore a blindfold during the infusion and the infusion port was kept out of sight. The recruitment process, the experimental measurements, and data analysis were conducted by an investigator who was blind to the nature of the infusion.

### 

#### Participants.

Twenty-two healthy participants were studied so as to be able to detect a difference of ∼4 mmHg in pulmonary artery pressure between the two groups at a significance level of *P* < 0.05 with a power of 80%. Participants were recruited by advertisement. Inclusion criteria required participants to be healthy and iron replete (ferritin 20–300 μg/l, transferrin saturation 20–50%) and to have detectable physiological tricuspid regurgitation on echocardiography to allow for measurement of PASP. Exclusion criteria were any significant comorbidity that may affect hematology parameters or iron status, or pulmonary vascular or ventilatory responses. Participants with recent exposure to an altitude >2,500 m or air travel >4 h within the week prior to any experimental day were also excluded.

Each participant gave informed written consent. The study was approved by the National Research Ethics Service Committee South Central–Oxford A (REC 12/SC/0586) and performed in accordance with the Declaration of Helsinki.

#### Iron infusion.

On *day 1*, participants received either 50 ml of sterile 0.9% m/V NaCl (placebo group) or 1 g of iron as ferric carboxylmaltose (Ferinject; Vifor Pharma, Glattbrugg, Switzerland) diluted in 50-ml sterile 0.9% m/V NaCl (iron group), administered over 15 min via a syringe infusion pump (Graseby 3100 Syringe Pump; Smiths Medical International, Ashford, UK). Ferric carboxymaltose has a half-life of 7.4–12.1 h (5).

#### Blood tests.

Venous blood samples were taken on each experimental day prior to any other interventions. A full blood count together with associated parameters was obtained through a hospital laboratory. Similarly, iron markers including the concentration of ferritin and transferrin, as well as transferrin saturation, were determined by a clinical biochemistry laboratory. In addition, enzyme-linked immunosorbent assays (ELISA) were performed in duplicate on serum to measure the concentration of EPO and interleukin 6 (IL-6) (Quantikine IVD human EPO and Quantikine high-sensitivity human IL-6; R&D Systems, Abingdon, UK) and on plasma to measure soluble transferrin receptor concentration (sTfR) (Quantikine IVD human sTfR; R&D Systems) and hepcidin concentration (Bachem, Peninsula Laboratories, Torrance, CA) according to the manufacturers' instructions.

#### Hypoxic exposures.

The 6-h isocapnic hypoxic exposures were conducted using a purpose-built chamber (9). The target end-tidal Po_2_ was 55 mmHg, and the target end-tidal Pco_2_ was the naturally occurring air-breathing value for each participant, as assessed at the beginning of each experimental day. Respired gas was sampled continuously via a fine nasal catheter held in place at the opening of the participant's nostril. The samples were continually analyzed for CO_2_ and O_2_, and the chamber gas composition was automatically adjusted at 5-min intervals so as to minimize the difference between the desired and measured end-tidal values for Pco_2_ and Po_2_. Participants spent no longer than 10 min in total outside of the chamber, e.g., for toilet breaks, over the course of a visit.

#### Assessment of PASP.

PASP was estimated using standard echocardiographic techniques (27, 31) and equipment Vivid i (GE Healthcare, Chalfont St Giles, UK). In brief, the minor (physiological) degree of tricuspid regurgitation present in our participants resulted in a retrograde jet of blood from the right ventricle to the right atrium during systole. The velocity of this jet of blood was measured using Doppler ultrasound, and from this the peak pressure gradient across the tricuspid valve was determined and PASP was calculated using the modified Bernoulli's equation, with a value of 5 mmHg assumed for right atrial pressure (7). PASP was measured hourly during the hypoxic exposures. One blinded investigator obtained the echocardiographic images used for PASP measurements and analyzed the data. A second blinded investigator separately analyzed a sample of the images to check consistency of the analysis.

#### Statistics.

Statistical analysis was conducted either using IBM SPSS Statistics version 22.0 or using the nlme package in R (version R3.1.0; Vienna, Austria). Comparisons were drawn either by using unpaired two-tailed Student *t*-tests or by using linear mixed-effects modeling with participants as a random factor. For the blood results, where there was one value per day, the fixed effects were group (saline or iron), day, and the interactive term between the two. For the measurements of PASP, the fixed effects were group (saline or iron), day, and “state” (normoxia or sustained hypoxia) (*hours 4*, *5*, *6*, where the measurements of PASP were relatively stable), together with interactive terms. Statistical significance was assumed at *P* < 0.05.

## RESULTS

All participants were healthy and iron replete, and their demographics and *day 0* venous blood analyses are given in Table 1. All infusions were well tolerated. There were no major adverse events.

**Table 1.** Participant demographics and baseline venous blood analyses

Variable (Normal Range)	Saline Group (*n* = 11)	Iron Group (*n* = 11)	*P* Value
Age, yr	26.3 ± 8.6	33.5 ± 9.9	0.09
BMI, kg/m^2^	24.3 ± 1.6	24.9 ± 2.1	0.51
Sex	Female, *n* = 4; male, *n* = 7	Female, *n* = 4; male, *n* = 7	
Hemoglobin (12.0–17.0), g/dl	13.9 ± 1.3	14.0 ± 0.9	0.81
Hematocrit (0.36–0.50)	0.42 ± 0.04	0.42 ± 0.03	0.77
Mean cell volume (83–105), fl	93.3 ± 3.8	90.8 ± 5.0	0.20
Erythropoietin, IU/l	8.1 ± 3.4	9.3 ± 3.0	0.38
Serum ferritin (10–300), μg/l	66 ± 40	55 ± 37	0.52
Serum transferrin (1.8–3.6), g/l	2.6 ± 0.4	2.7 ± 0.3	0.44
Serum transferrin saturation (16–50), %	31.3 ± 11.3	29.3 ± 12.7	0.70
Hepcidin, μg/l	51.5 ± 57.9	40.2 ± 34.2	0.58
sTfR, nmol/l	20.6 ± 5.9	21.5 ± 6.1	0.73
Interleukin-6, fg/l	0.56 ± 0.41	0.56 ± 0.4	0.97
C-reactive protein (0–8), mg/l	0.8 ± 0.6	2.5 ± 3.2	0.30

Values are means ± SD. An unpaired two-tailed Student's *t*-test was used to calculate the *P* value. BMI, body mass index; sTfR, soluble transferrin receptor.

### 

#### Hematology variables.

**Fig. 2.**
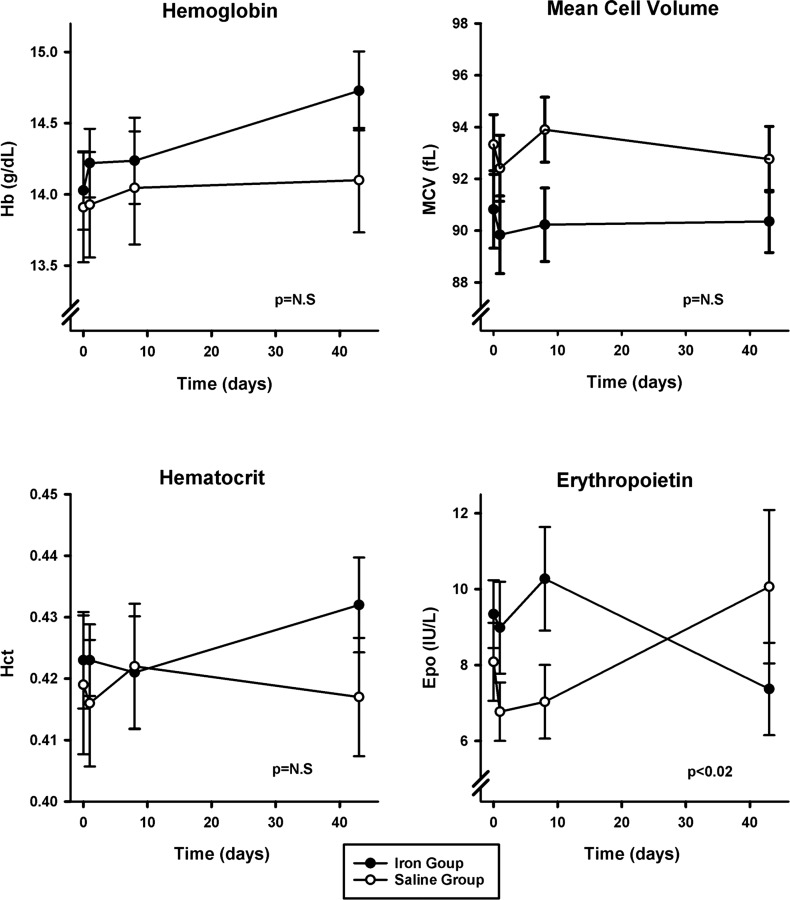
Hematology results. No significant effects of infusion of 1 g of iron were detected in hemoglobin concentration (Hb), hematocrit (Hct), or mean cell volume (MCV). Erythropoietin concentration (EPO) appeared mildly elevated at *day 8* following iron infusion compared with controls. Values and error bars are means ± SE. *P* values refer to the interactive term for the change over time between groups and are calculated using linear mixed-effects modeling. N.S, not significant.

The two groups were well matched at *day 0* for hemoglobin concentration, hematocrit, and mean cell volume (Table 1). These did not vary differentially over time between the saline and iron groups (Fig. 2). The groups were similarly well matched for EPO concentration at *day 0* (Table 1), but these values diverged such that EPO appeared higher at *day 8* in the iron group compared with control (*P* < 0.02) (Fig. 2).

#### Assessment of iron status.

**Fig. 3.**
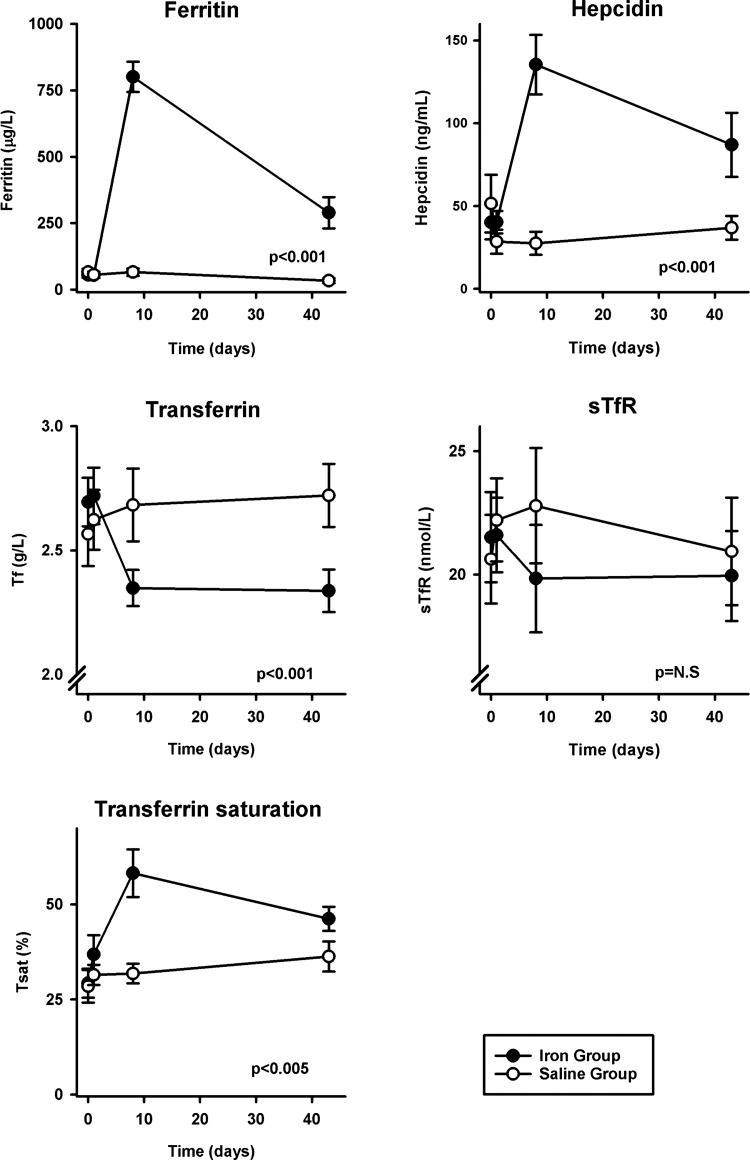
Iron indexes. Following infusion of 1 g of iron, there were significant increases in the concentration of ferritin, transferrin saturation (Tsat), and hepcidin and a significant fall in transferrin concentration (Tf). No significant effects were observed in soluble transferrin receptor concentration (sTfR). Ferritin *n* = 6 for both groups at *day 43*. Values and error bars are means ± SE. *P* values refer to the interactive term for the change over time between groups and are calculated using linear mixed-effects modeling.

Values for the concentration of ferritin, transferrin, transferrin saturation, hepcidin, and sTfR were well matched between the saline and iron groups at *day 0* (Table 1). Iron loading with 1 g of iron led to an acute rise in ferritin concentration (*P* < 0.001), transferrin saturation (*P* < 0.005), and hepcidin concentration (*P* < 0.001) by *day 8* (Fig. 3). These changes remained significant at *day 43*. Transferrin concentration fell post-iron infusion and had not returned to preinfusion levels by *day 43* (*P* < 0.001) (Fig. 3). Values for sTfR concentration did not differ significantly over time between the saline and iron infusion groups (Fig. 3).

#### Gas control during sustained hypoxic exposures.

**Fig. 4.**
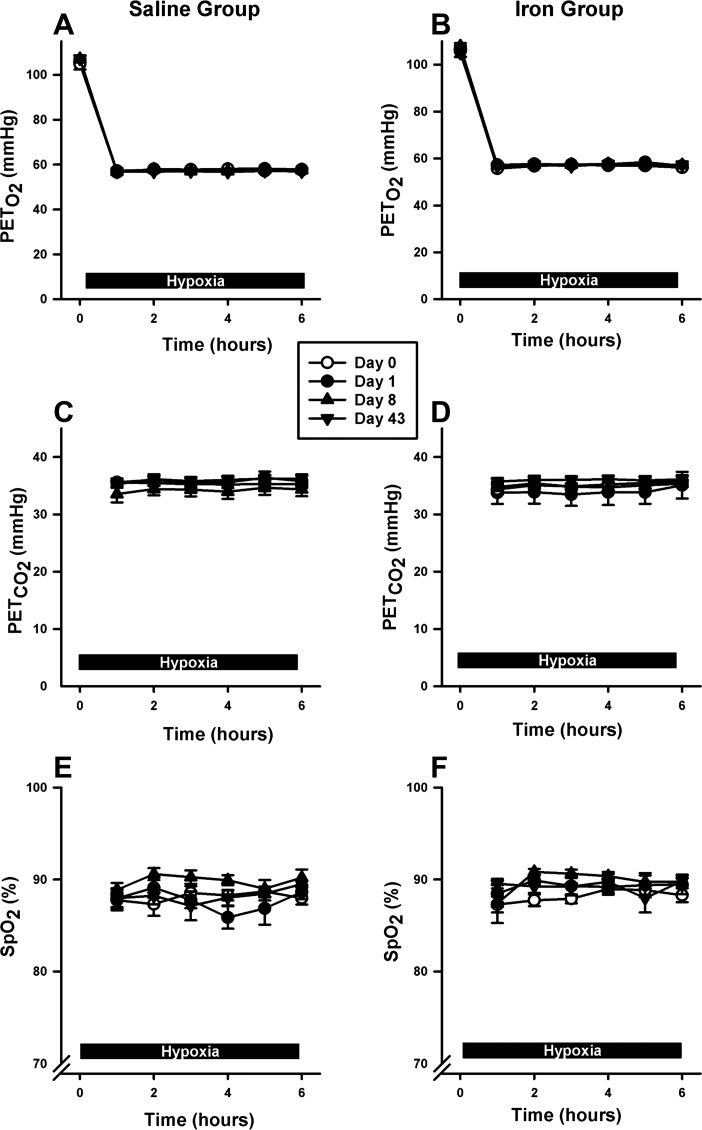
Gas control during exposure to hypoxia. *A* and *B*: end-tidal oxygen (Pet_O_2__) levels over 6 h for saline and iron groups, respectively. *C* and *D*: end-tidal carbon dioxide (Pet_CO_2__) levels for saline and iron groups, respectively. *E* and *F*: arterial hemoglobin saturation (SpO_2_) for saline and iron groups, respectively. Values and error bars are means at each hour of hypoxia ± SE.

Values for end-tidal Pco_2_ and Po_2_ were well matched between the two groups during the hypoxic exposures in the chamber on each experimental day (Fig. 4). Similarly, average saturation of oxygen measured by pulse oximetry was well matched between the two groups across experimental days (Fig. 4).

#### PASP response to hypoxia.

**Fig. 5.**
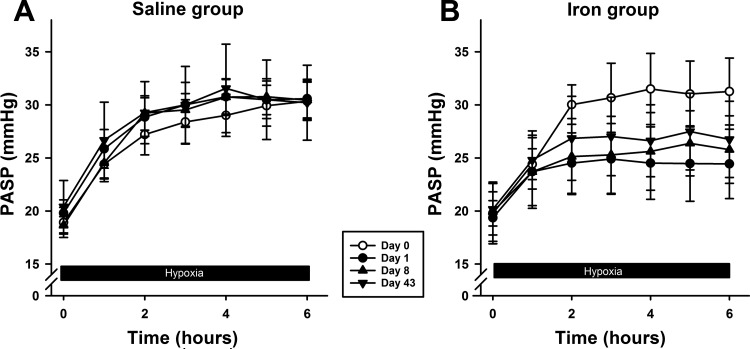
Pulmonary artery systolic pressure (PASP) during exposure to hypoxia. *A* and *B:* PASP responses in saline and iron groups, respectively. Responses to sustained hypoxia were blunted following iron infusion on *day 1* and remained blunted at *days 8* and *43* (*P* < 0.001). Values and error bars are means ± SE.

The responses of PASP to hypoxia are illustrated in Fig. 5. A progressive rise in PASP was observed in all participants over the 6-h period of hypoxia. The interactive terms between hypoxia and group on *days 1*, *8*, and *43* were all significantly different from *day 0* (each *P* < 0.001). The interactive term between hypoxia and group for *day 0* (before iron was administered) was not significant. Thus iron significantly attenuated the PASP response to hypoxia, by ∼50% on *day 1*, and the response then remained suppressed over the 6-wk study period.

## DISCUSSION

The main finding of this study is that the suppression of the pulmonary vascular response to sustained hypoxia following IV administration of iron persists at 8 and 43 days after administration. This finding demonstrates that the effects of iron administration on the pulmonary vasculature are not simply due to the presence of a foreign iron-sugar complex in the blood, as this is long eliminated by the 43-day time point (5). Rather, the effect persists after the iron dose has been incorporated into the body's stores of iron.

Total body iron in a normal iron-replete individual is ∼4 g (15). Therefore the dose of 1 g of IV iron represents an elevation of total body iron of ∼25%. The loss of iron from the body, mainly from the gut, is ∼1–2 mg/day (15), and in the absence of either further iron absorption or any additional iron loss, it will take ∼2–3 yr for iron stores to return to preinfusion values. In our study, apart from sTfR (itself a marker of cellular iron demand rather than iron overload), values for the concentration of plasma ferritin, transferrin, and transferrin saturation all remained altered at 8 and 43 days post-iron infusion. What is not clear is whether any, or all, of these indexes of iron status will return to normal somewhat more rapidly than the rate at which the additional iron is lost from the body or whether they will only return to preinfusion values once the additional iron is lost.

A similar uncertainty relates to the pulmonary vascular response to hypoxia. It is uncertain whether this response is related to one or more of the circulating indexes of iron status or whether it is linked more directly to overall iron stores. From a more clinical perspective, the real interest is that effects of iron administration on the pulmonary vasculature are persistent; they last for a long time after the iron-sugar compound has been eliminated from the bloodstream. Thus the iron stores of the body could potentially be manipulated artificially to derive therapeutic gain.

Our underlying hypothesis for investigating the effects of iron on the pulmonary vasculature has been that iron availability affects the abundance of the transcription factor HIF, which itself is implicated in the pulmonary vascular response to hypoxia. While there is a very high degree of certainty both that iron modulates the HIF pathway (1, 13, 36) and that the HIF pathway affects the pulmonary vascular response to hypoxia (3, 8, 27, 38), it nevertheless cannot be concluded with equivalent certainty that the actions of iron availability on HIF are indeed the mechanism by which iron affects the pulmonary vascular response to hypoxia.

An interesting observation in our study is that the effects of iron on PASP become apparent only after the first hour of sustained hypoxia, and not at 1 h or before (Fig. 5). This finding is consistent with previous work (33). Thus iron does not appear to affect the acute (within minutes) hypoxic pulmonary vascular response, but rather the second phase of intensification of hypoxic pulmonary vasoconstriction, which begins after ∼45 min and causes a further progressive rise in PASP (32). This second slower phase of intensification has been attributed to new gene expression triggered by hypoxia (33), which may well be HIF regulated.

If indeed iron is working through HIF, then we may reasonably expect other HIF-regulated aspects of biology to be affected by manipulation of iron stores. For example, in humans it has been shown that iron chelation increases plasma EPO (20). However, in the present study, iron supplementation also appeared to increase EPO (at the *day 8* time point), perhaps via a separate IRP-related effect on HIF2α (6). Furthermore, there have been no reports of iron manipulation affecting the ventilatory response to sustained hypoxia despite the fact that HIF has been implicated in this process (12, 25, 27). This is similar to the PHD inhibitors, which have been shown to have a marked effect on erythropoiesis but to have minimal effects on the ventilatory response (2). Biologically, there are many complex issues to consider. First, manipulation of iron stores may have much greater effects on some cell types than others. Therefore the effects of manipulating the HIF pathway through iron stores may be specific to certain cell types. Second, HIF1 and HIF2 quite clearly have different actions (14, 19), and therefore it may be the case that iron has differential effects in relation to these two isoforms (11, 30). Third, although the classical effect of iron on the HIF pathway is through its involvement in the catalytic hydroxylation of HIF by the PHD enzymes, it has also been shown that cellular iron status affects the translation of HIF2α through the presence of an IRE in the 5′ end of the HIF2α mRNA (6, 24, 37). Again, this is a potential mechanism that could underlie both cell-specific and isoform-specific actions of iron on the HIF pathway.

## GRANTS

The research was funded by the National Institute for Health Research (NIHR) Oxford Biomedical Research Centre Programme. The views expressed are those of the author(s) and not necessarily those of the NHS, the NIHR, or the Department of Health. N. K. Bart was supported by the Sir John Monash Scholarship and Avant Scholarship. N. Petousi is supported by a NIHR Clinical Lectureship.

## DISCLOSURES

P. A. Robbins has received funding for basic science studies on iron homeostasis from Vifor Pharma. M. K. Curtis has received salary support from these funds.

## AUTHOR CONTRIBUTIONS

N.K.B., M.K.C., H.-Y.C., S.L.H., R.M., and K.L.D. performed experiments; N.K.B., M.K.C., H.-Y.C., R.M., and N.P. analyzed data; N.K.B., M.K.C., N.P., K.L.D., and P.A.R. interpreted results of experiments; N.K.B. and P.A.R. prepared figures; N.K.B. and P.A.R. drafted manuscript; N.K.B., M.K.C., H.-Y.C., S.L.H., R.M., N.P., K.L.D., and P.A.R. approved final version of manuscript; M.K.C., H.-Y.C., N.P., K.L.D., and P.A.R. edited and revised manuscript; P.A.R. conception and design of research.
